# Supplementary figures and images for: IκB kinase phosphorylates cytoplasmic TDP-43 and promotes its proteasome degradation
Source: J Cell Biol. 2024 Jan 10;223(2):e202302048. doi: 10.1083/jcb.202302048 (PMC10783433; doi:10.1083/jcb.202302048)

C

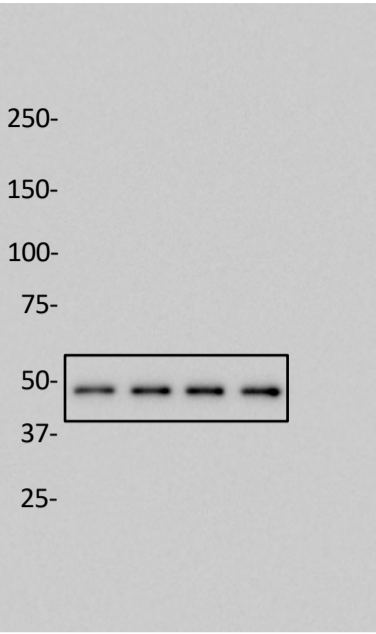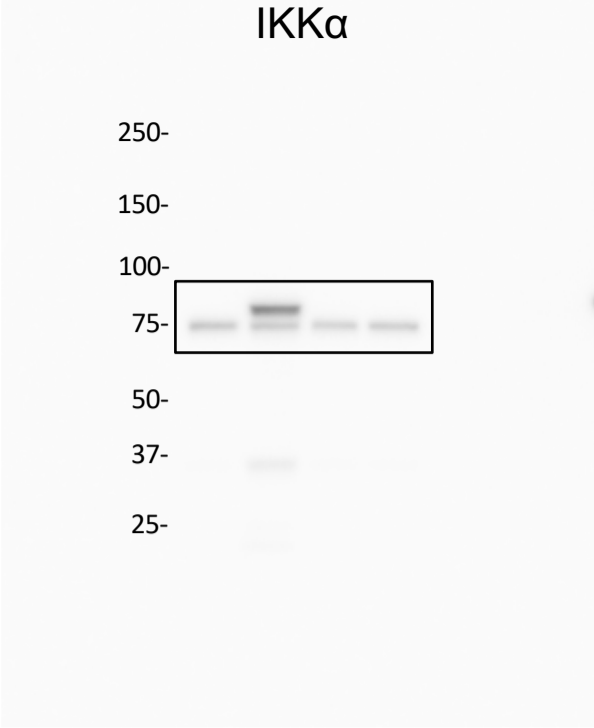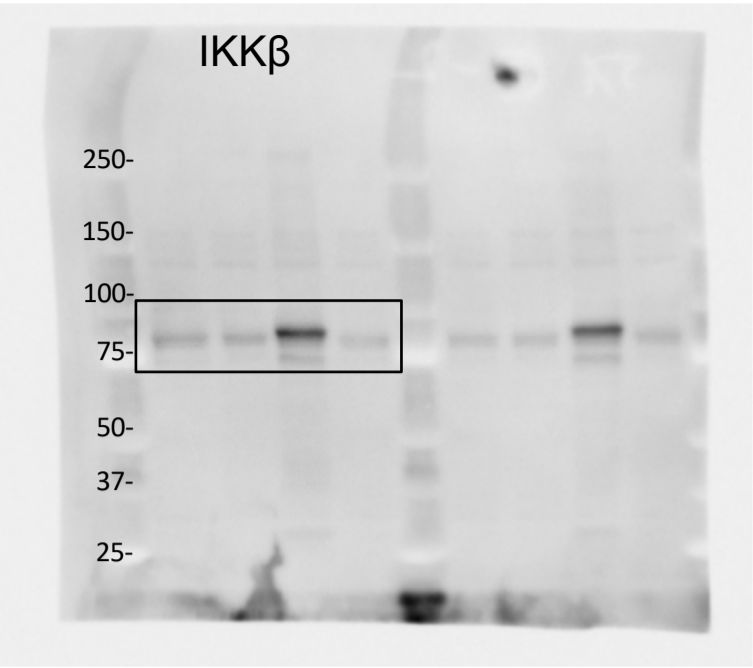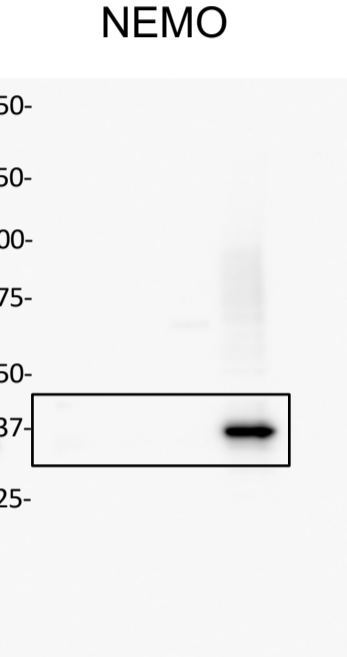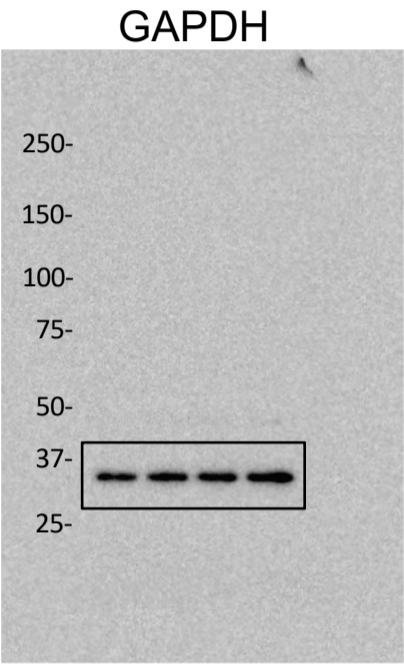

F

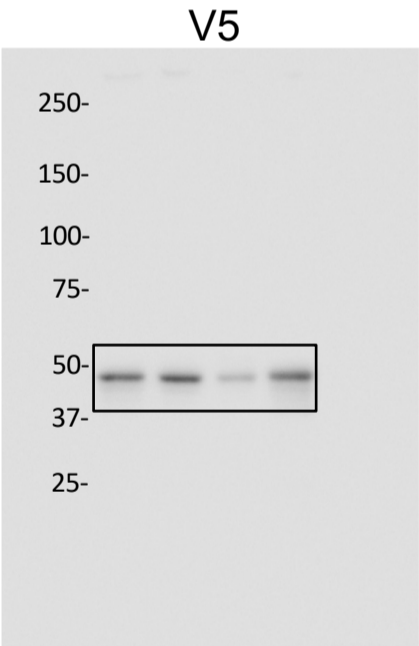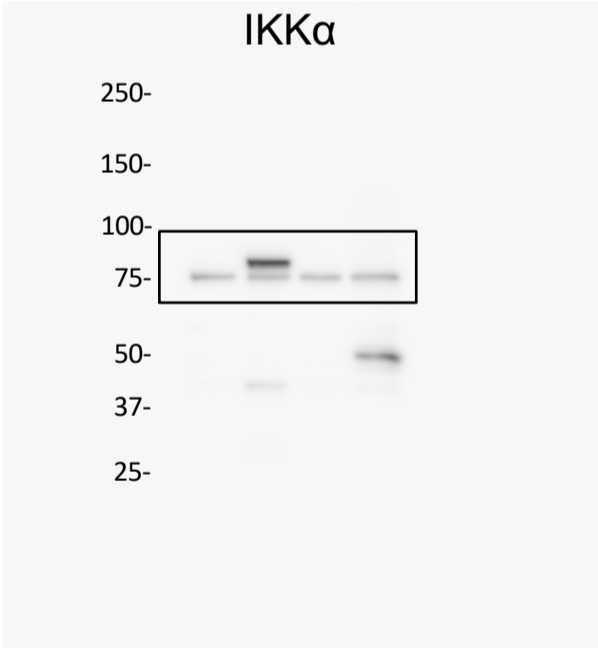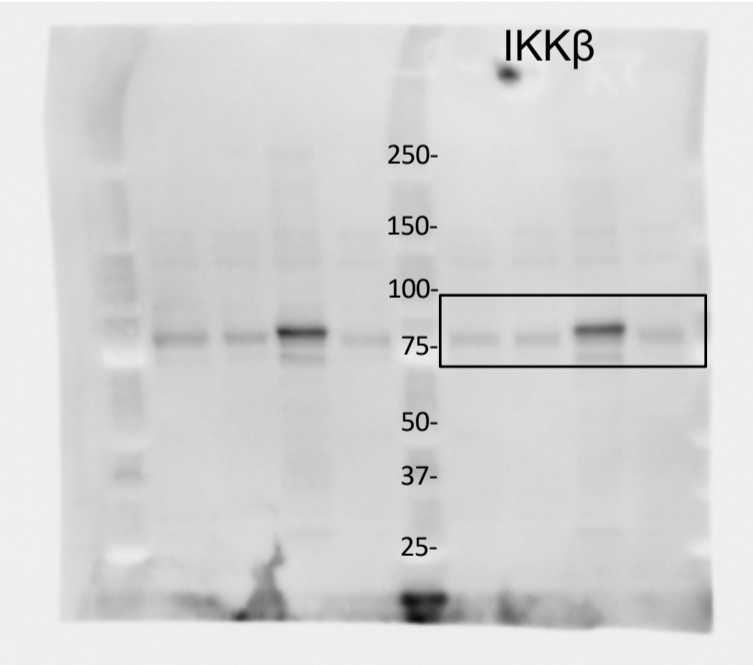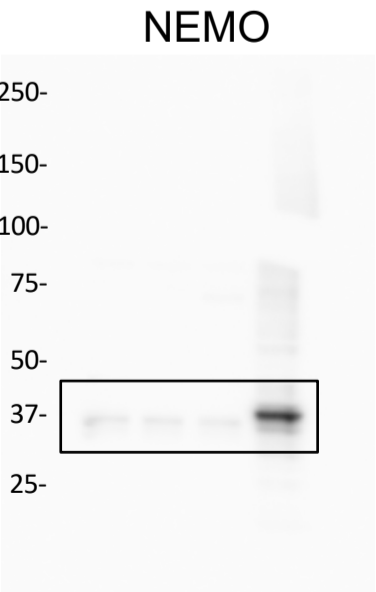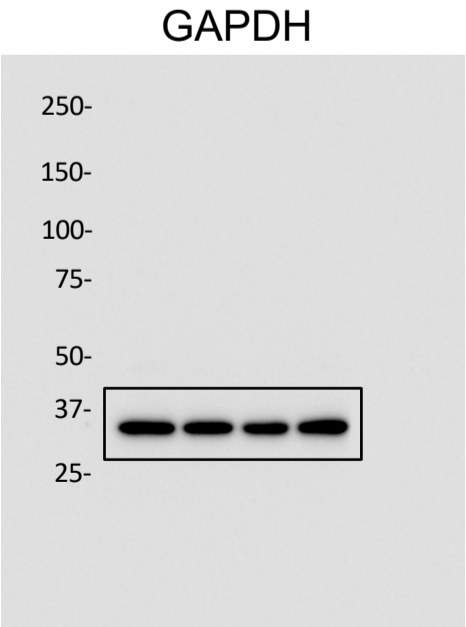

Supplement: Supplementary file 3 — SourceData F1 is the source file for Fig. 1. [file JCB_202302048_SourceDataF1.pdf]

B

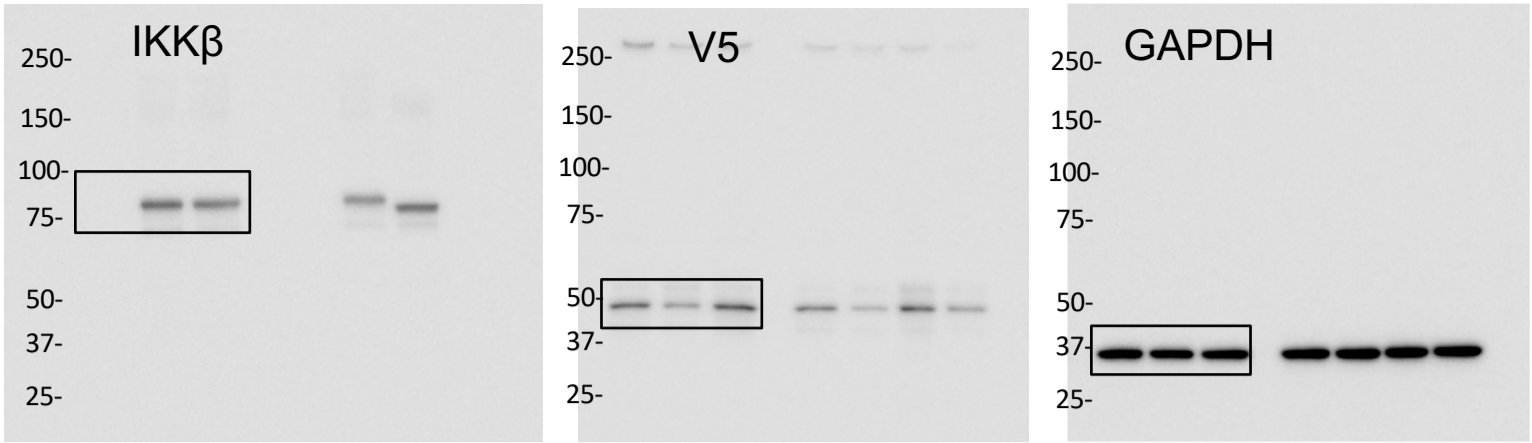

E

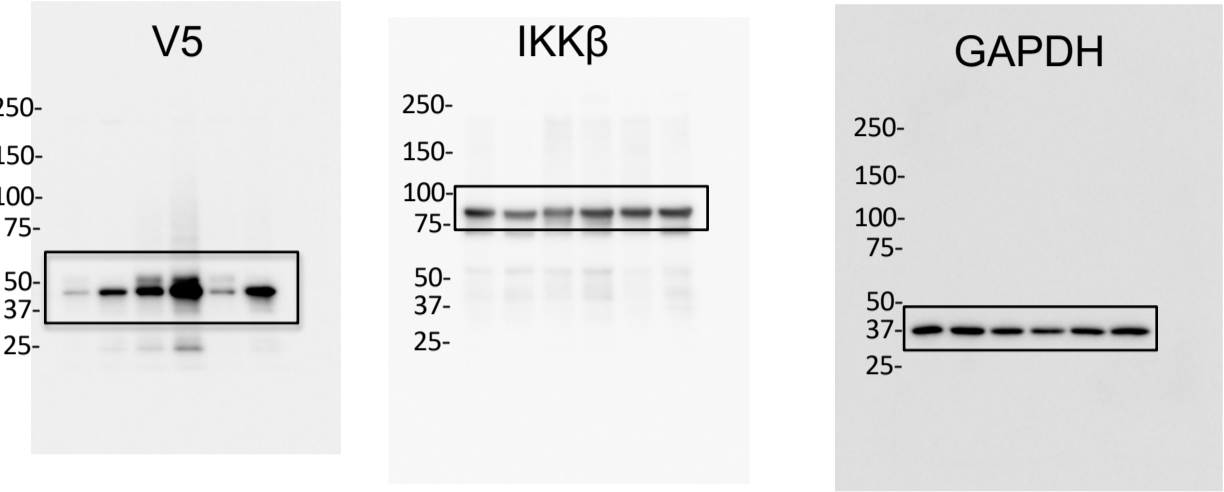

H

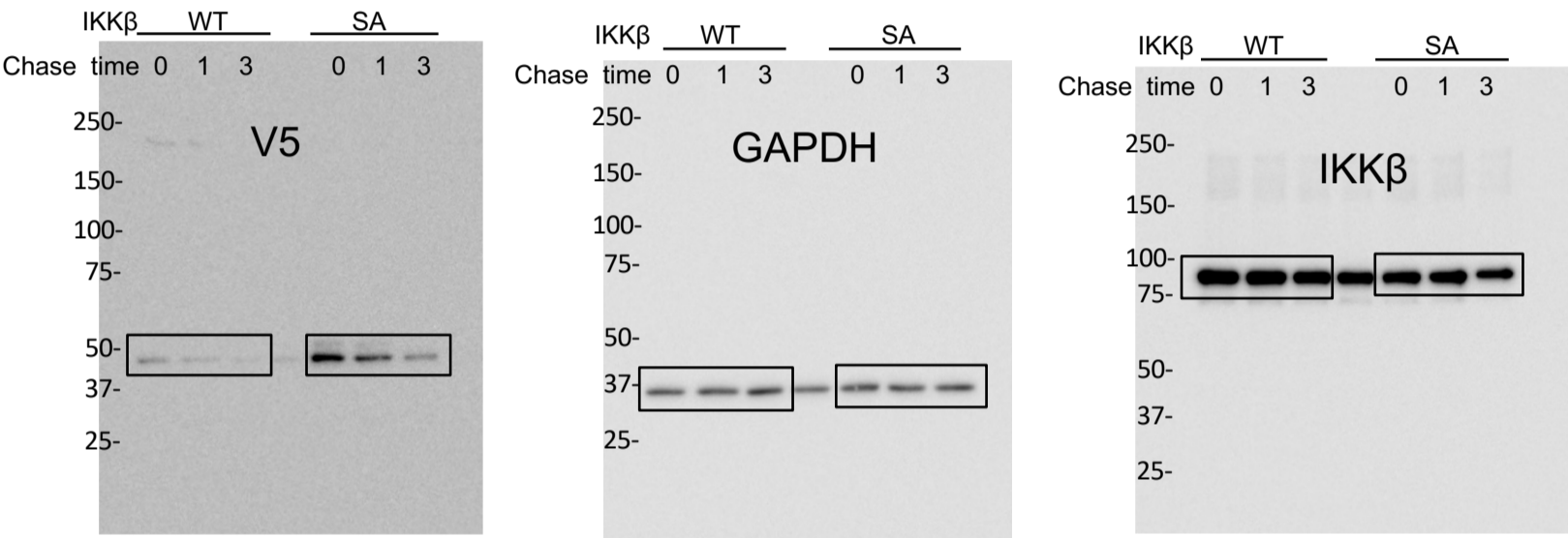

J

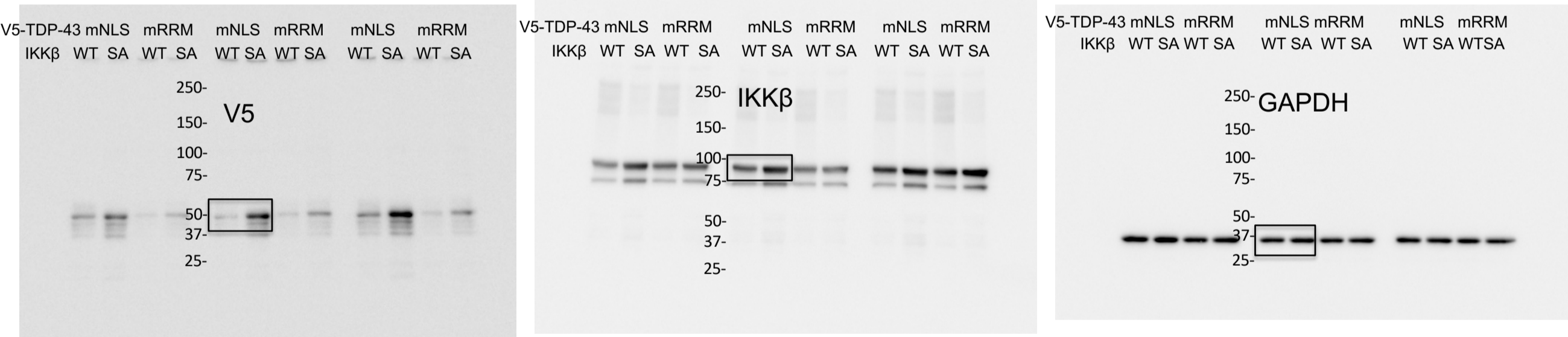

L

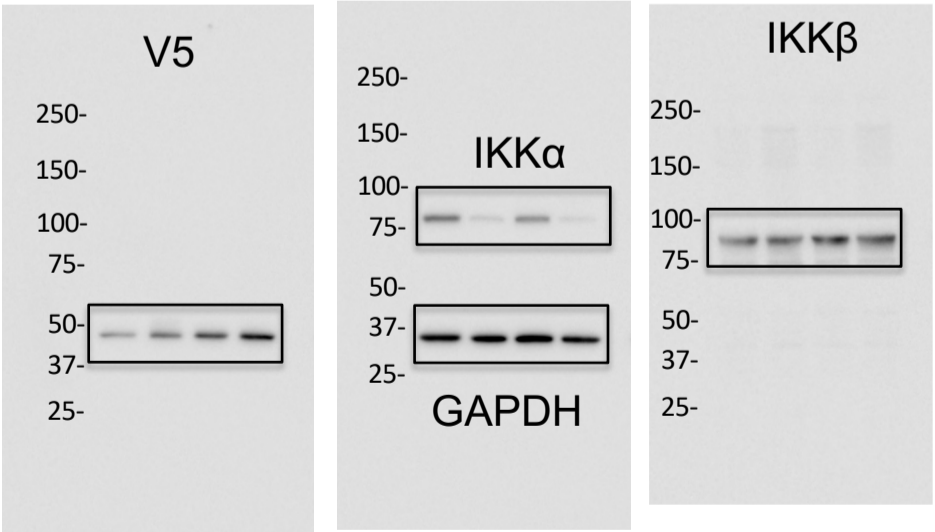

P

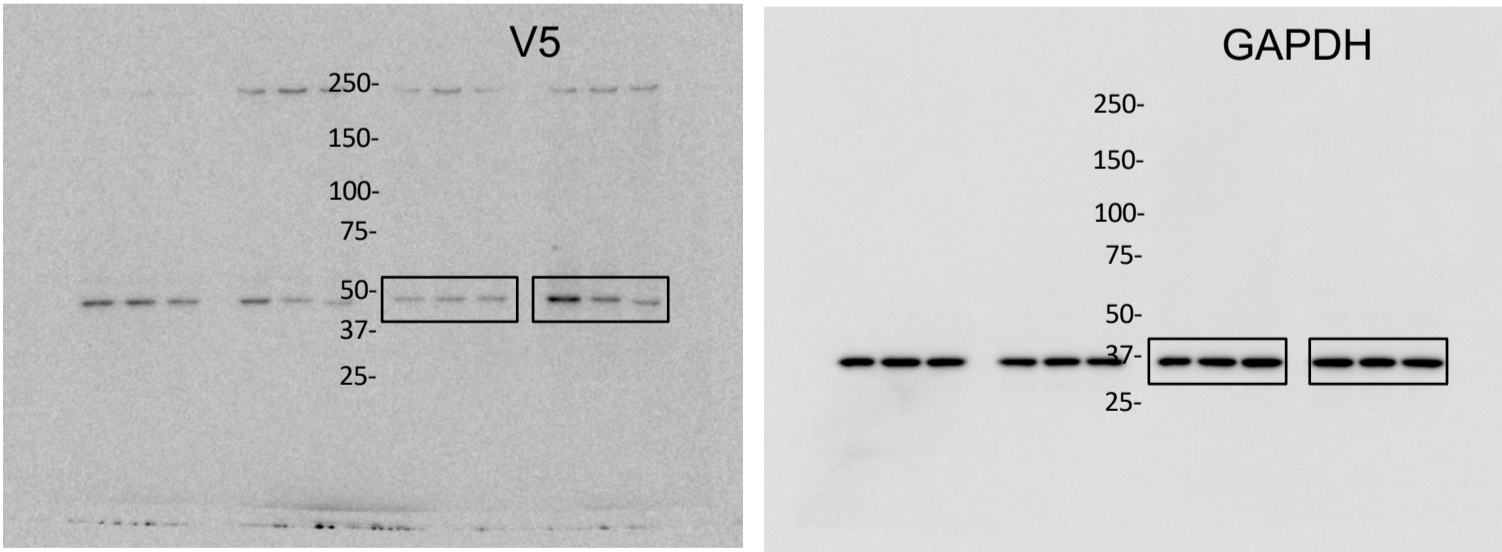

Supplement: SourceData F2 — is the source file for Fig. 2. [file JCB_202302048_SourceDataF2.pdf]

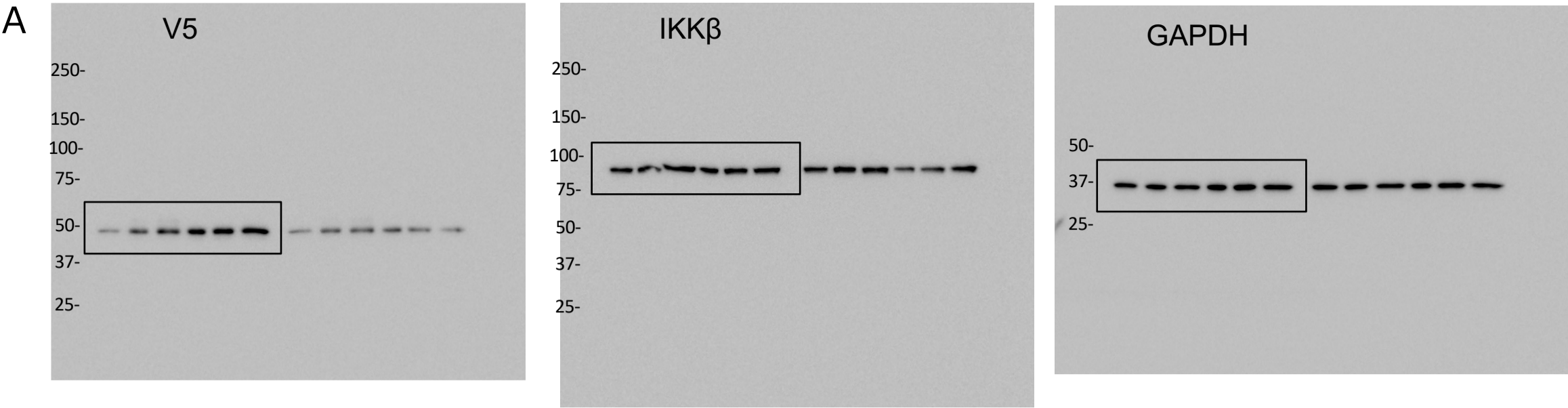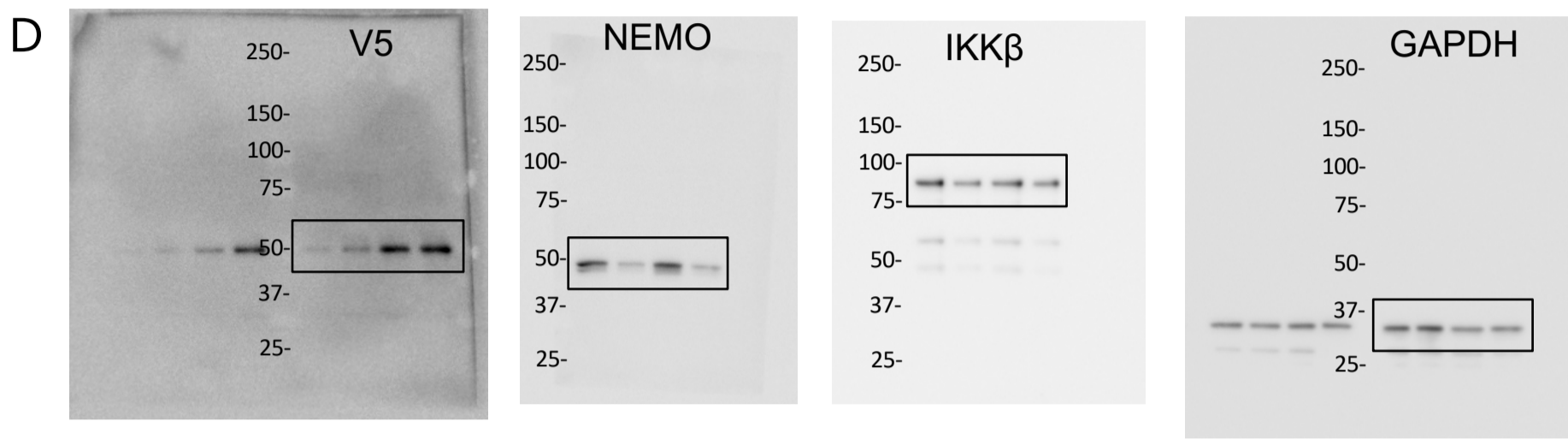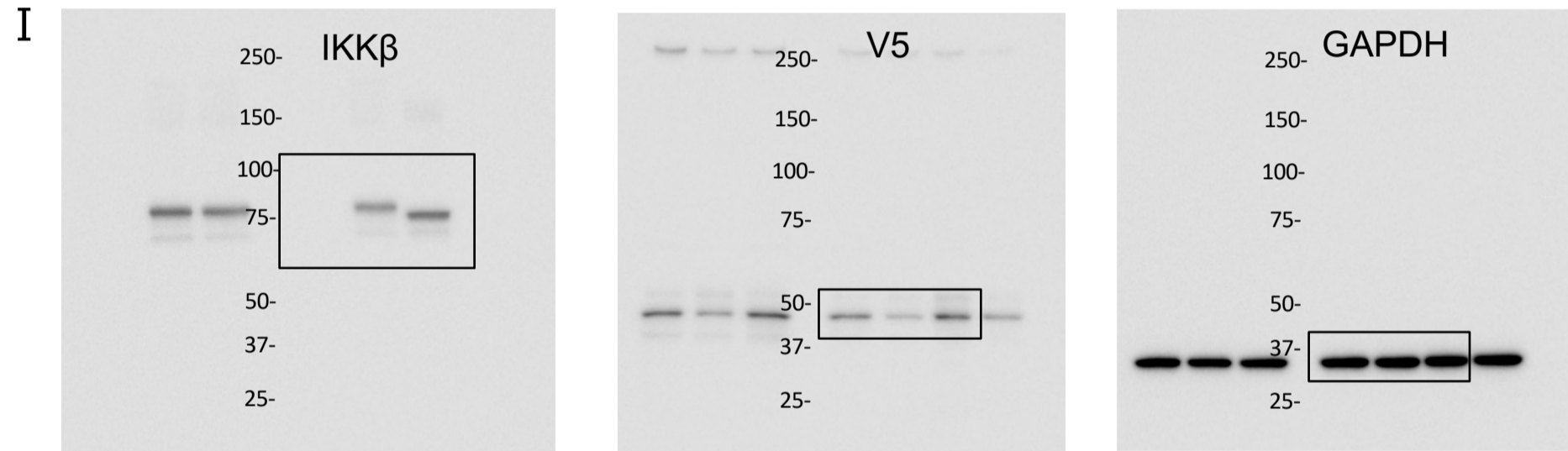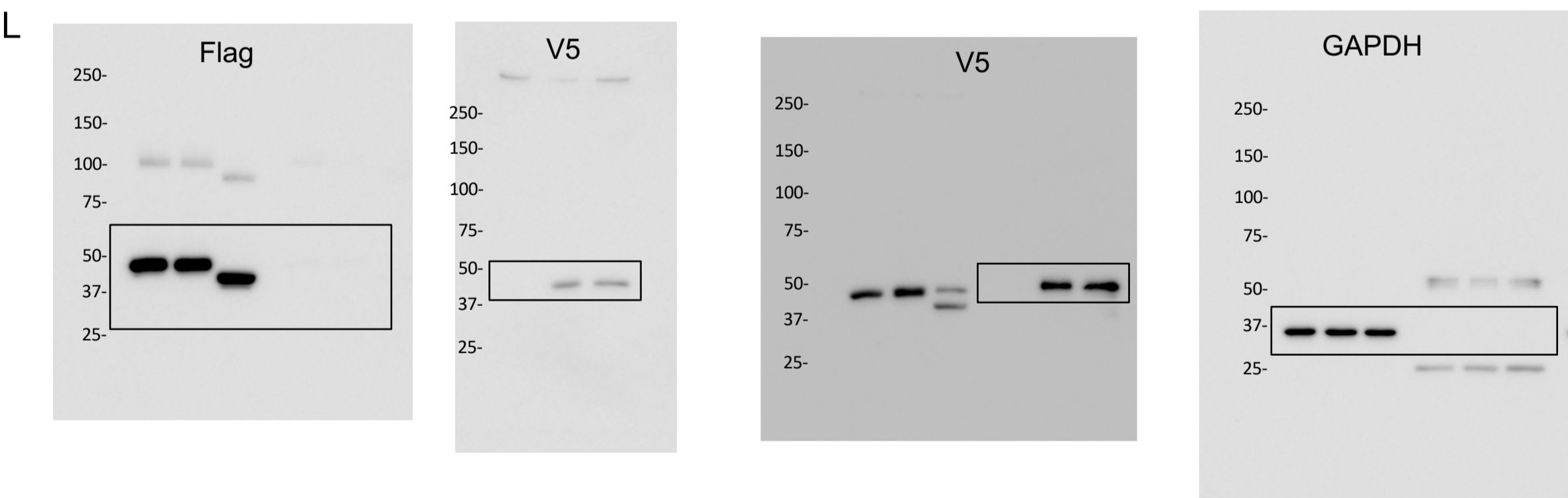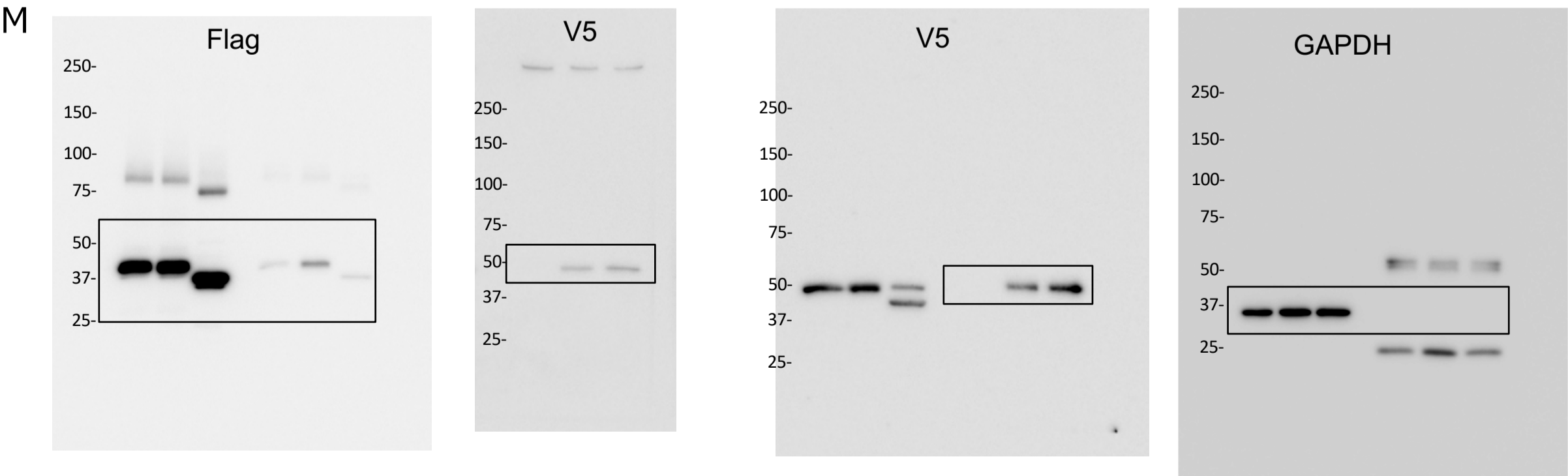

Supplement: SourceData F3 — is the source file for Fig. 3. [file JCB_202302048_SourceDataF3.pdf]

A

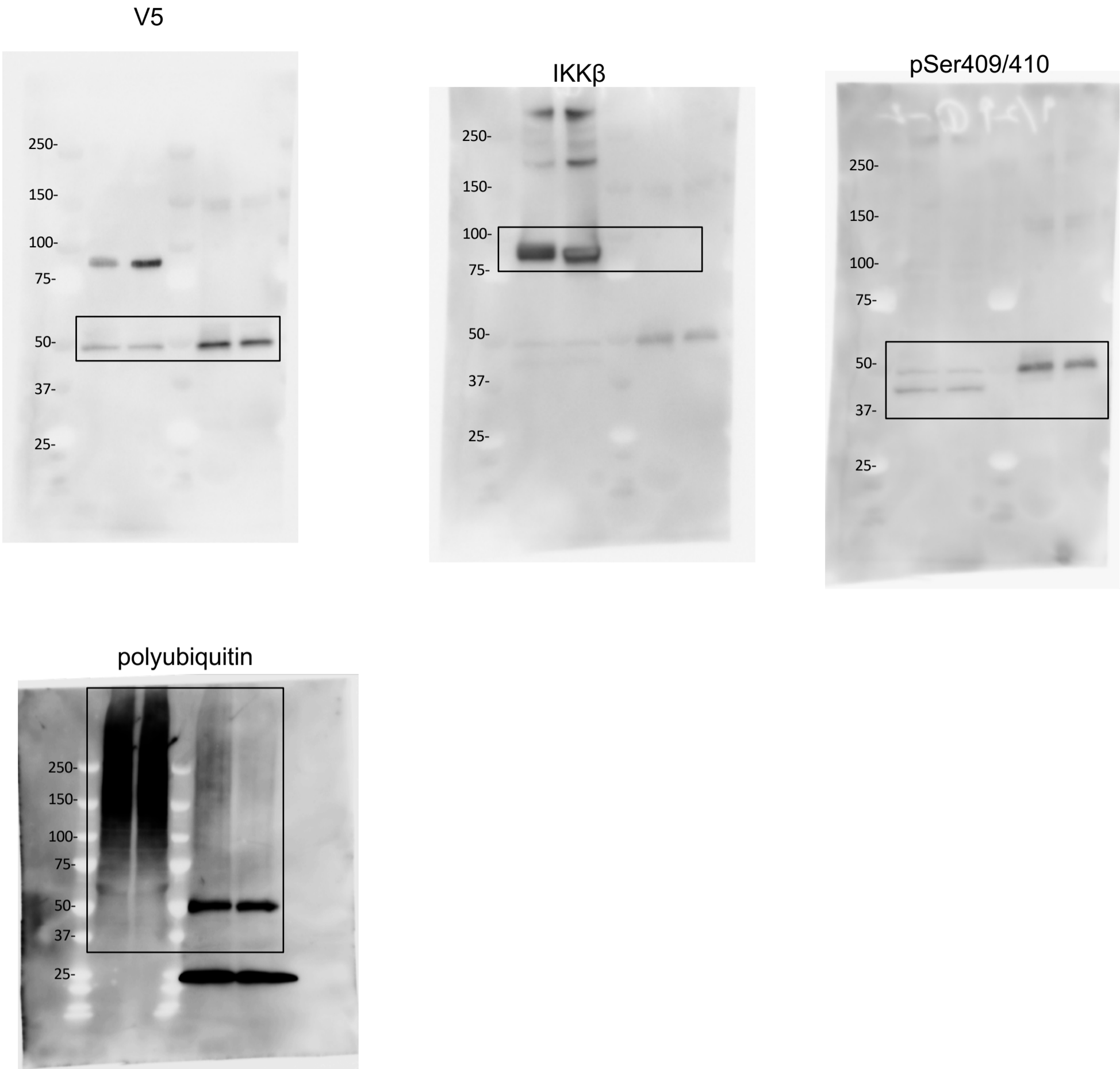

Supplement: SourceData F4 — is the source file for Fig. 4. [file JCB_202302048_SourceDataF4.pdf]

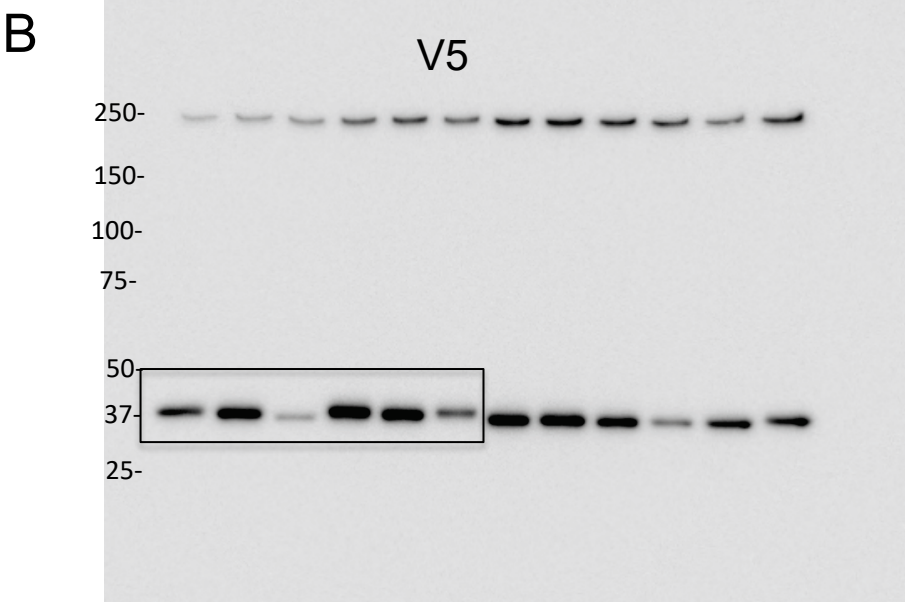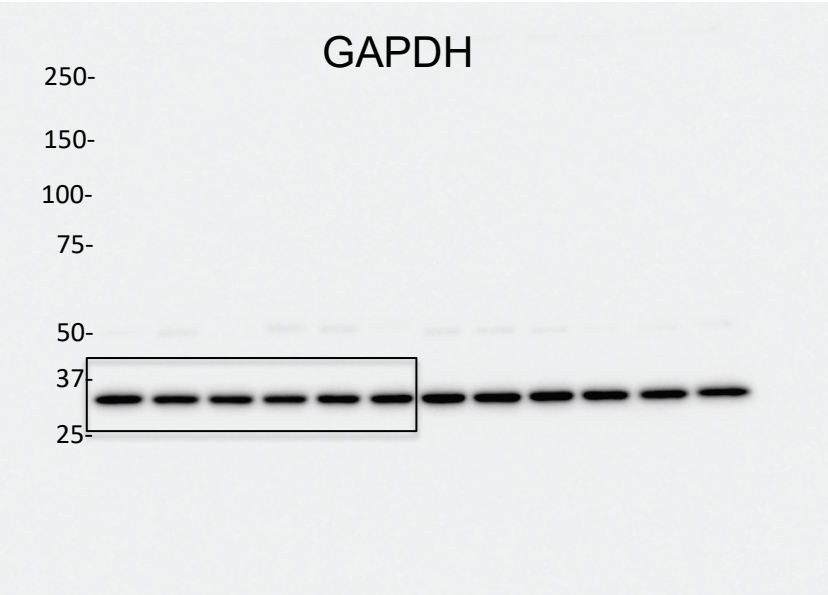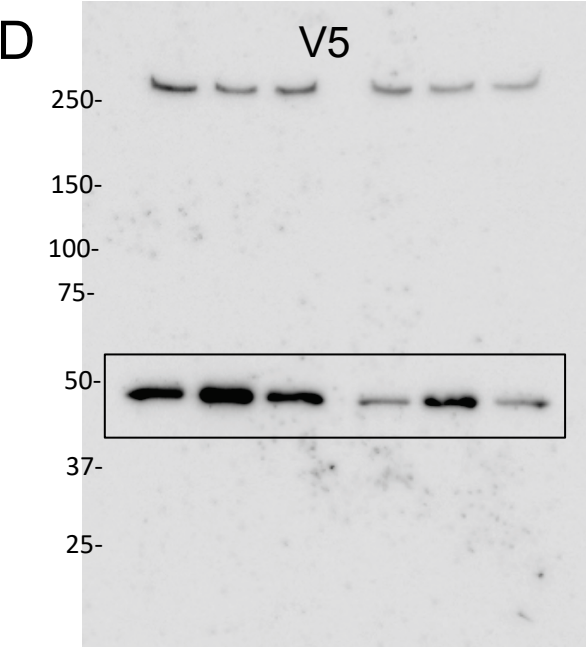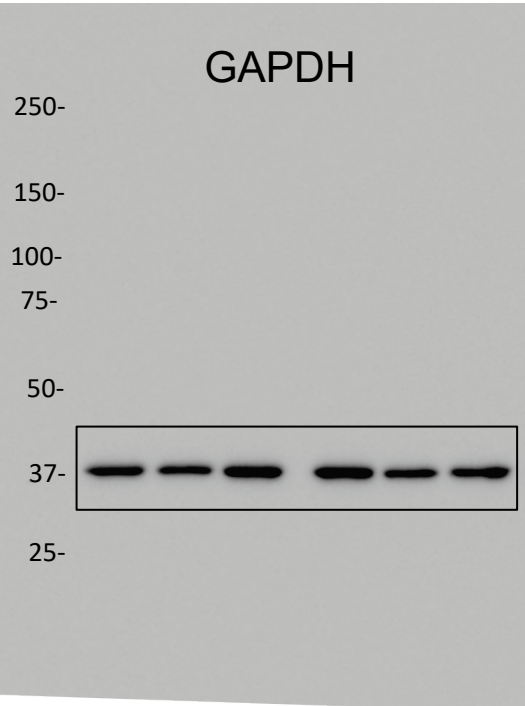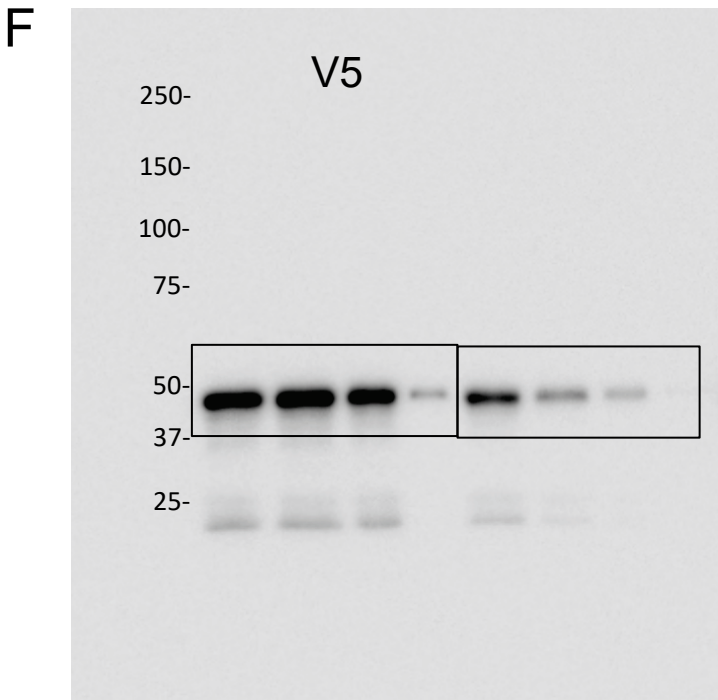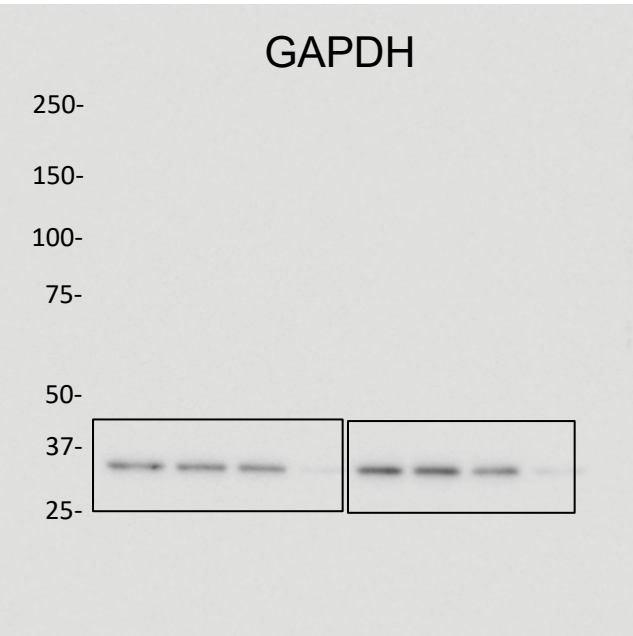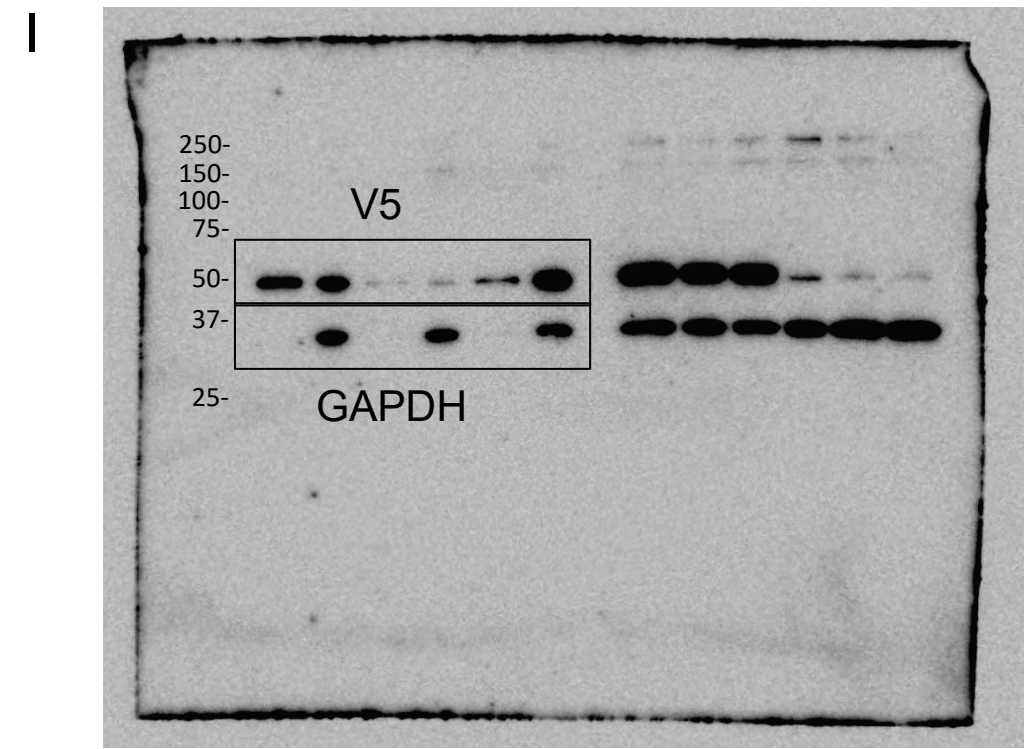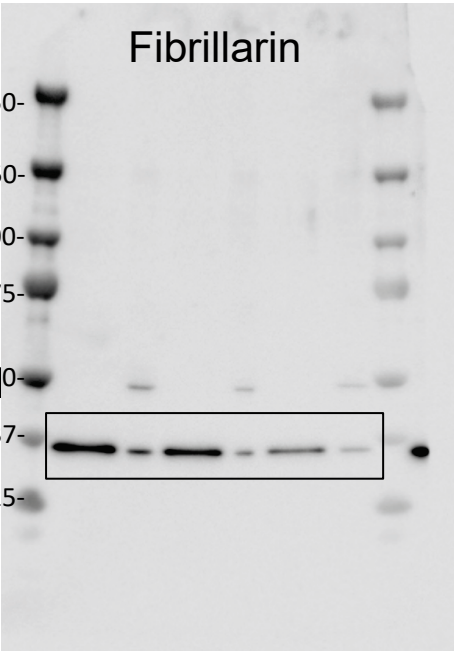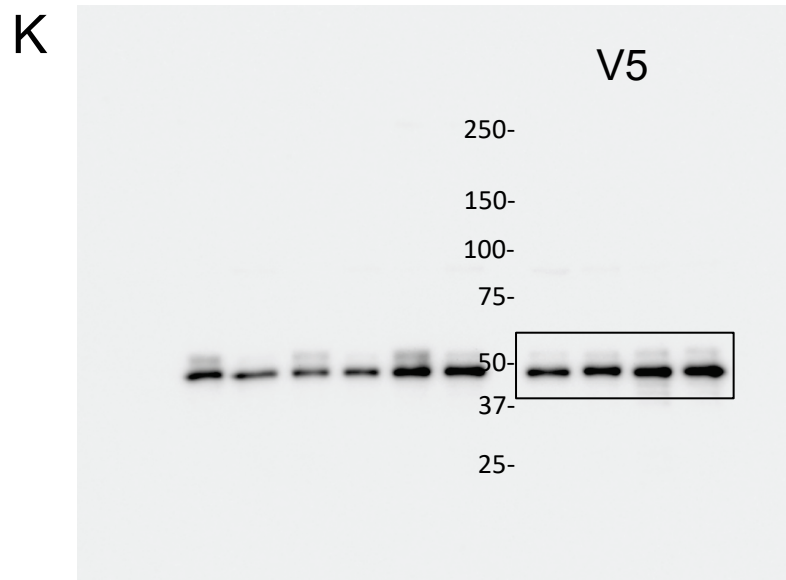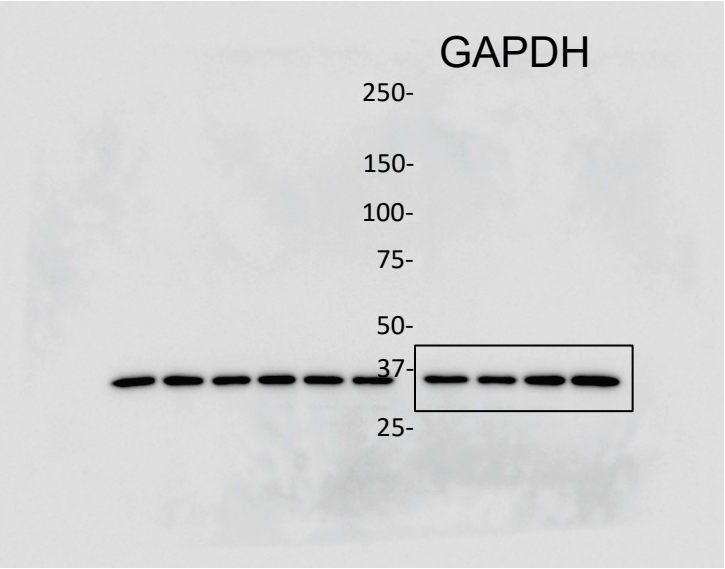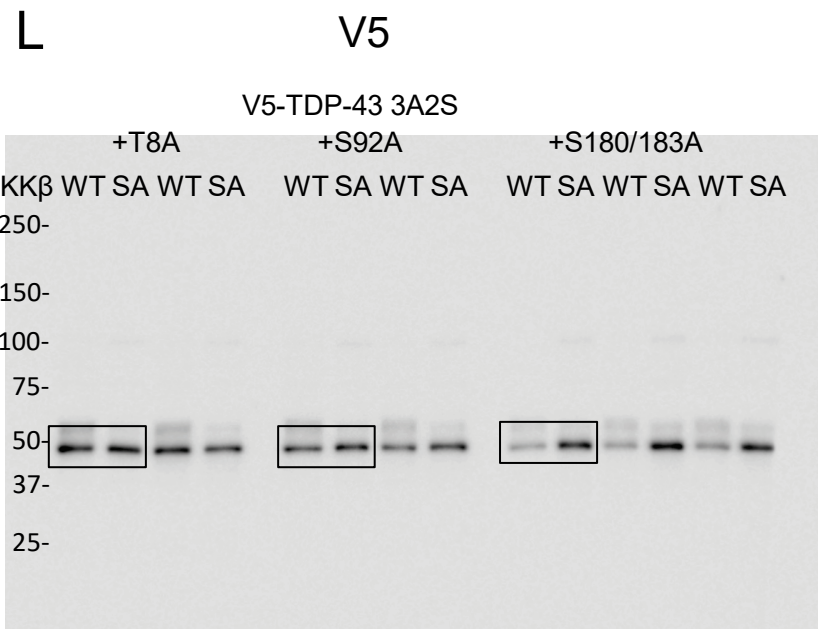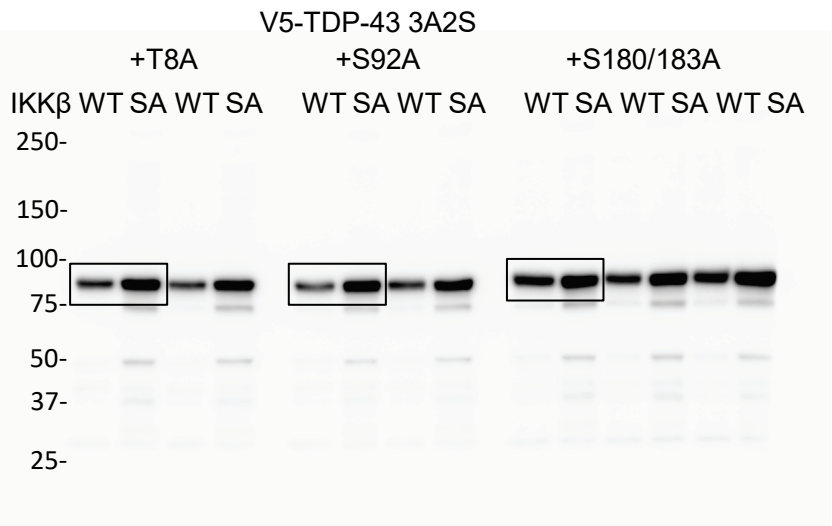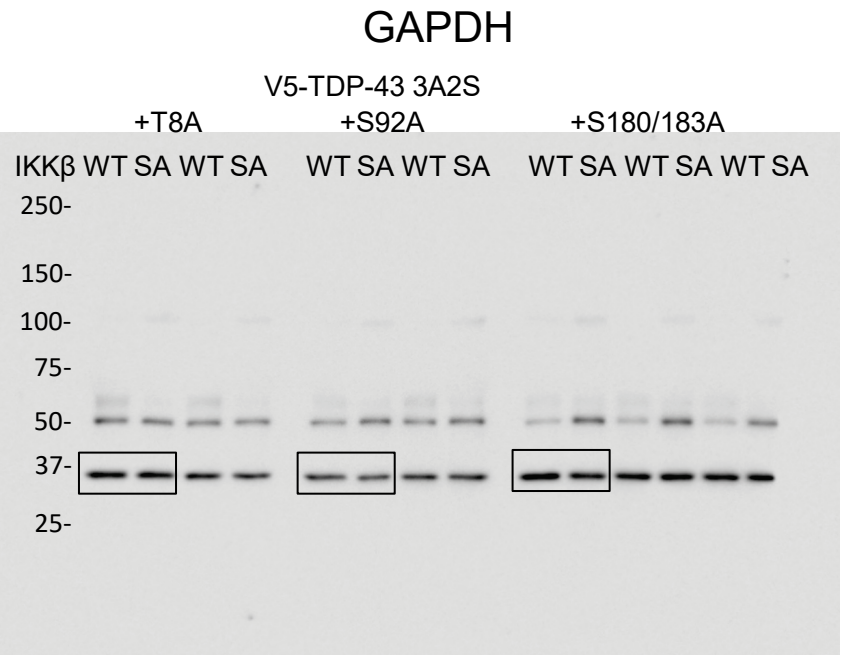

Supplement: SourceData F5 — is the source file for Fig. 5. [file JCB_202302048_SourceDataF5.pdf]

Because stripping and reblotting did not work well, the lysates were blotted again.

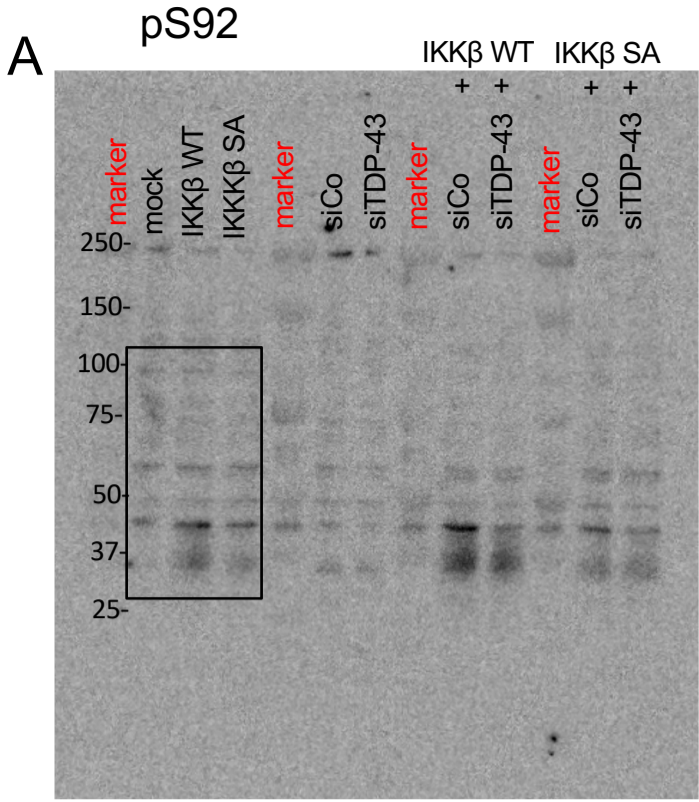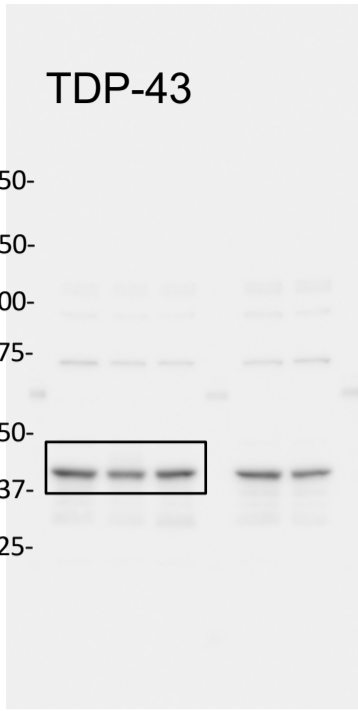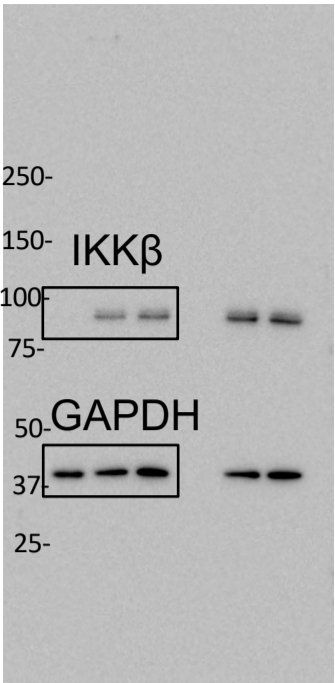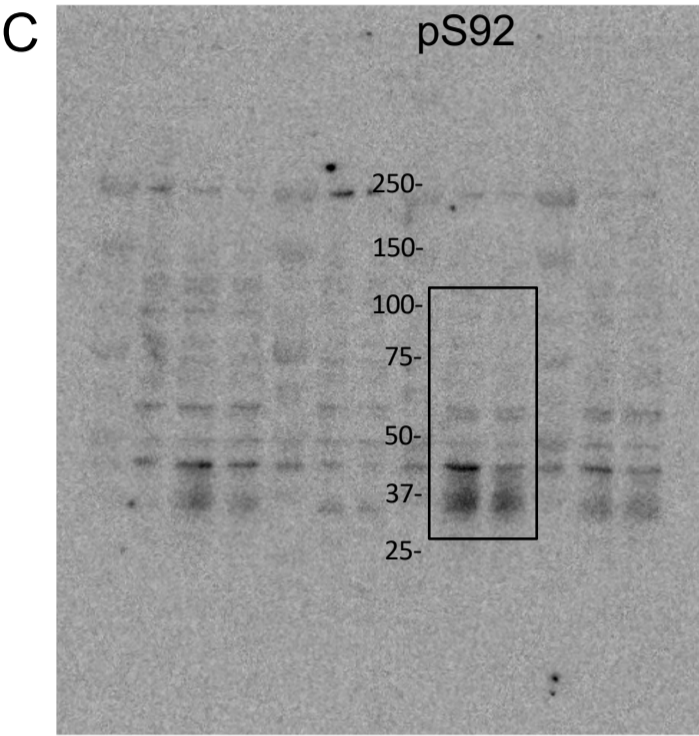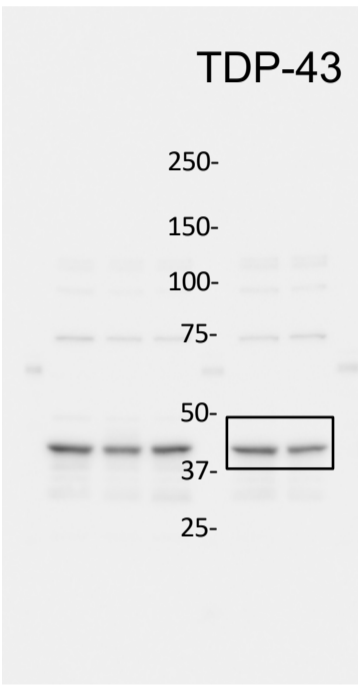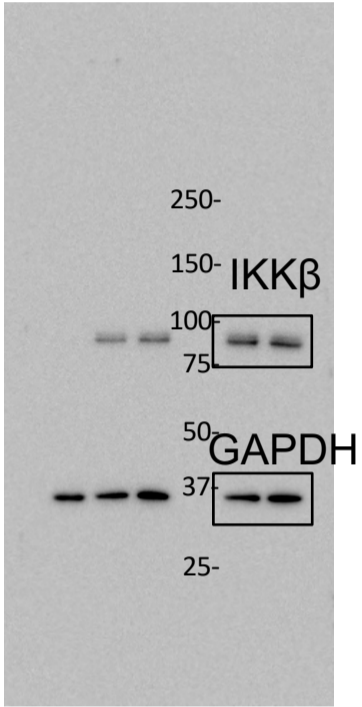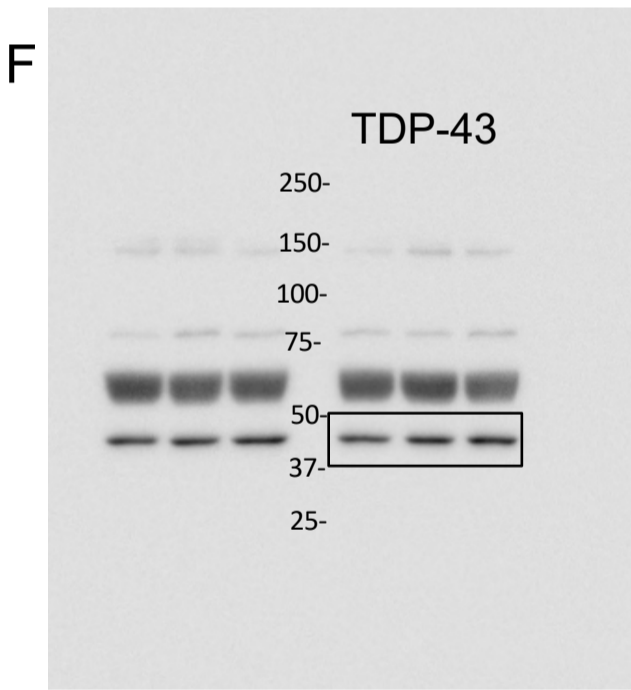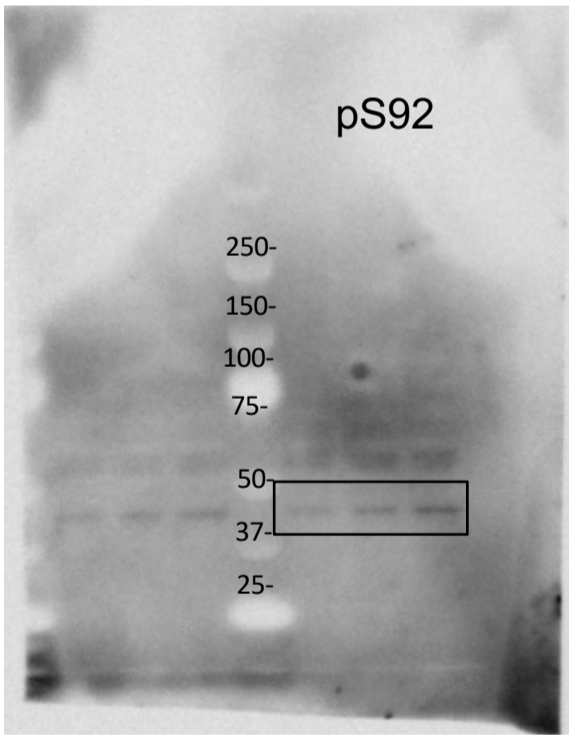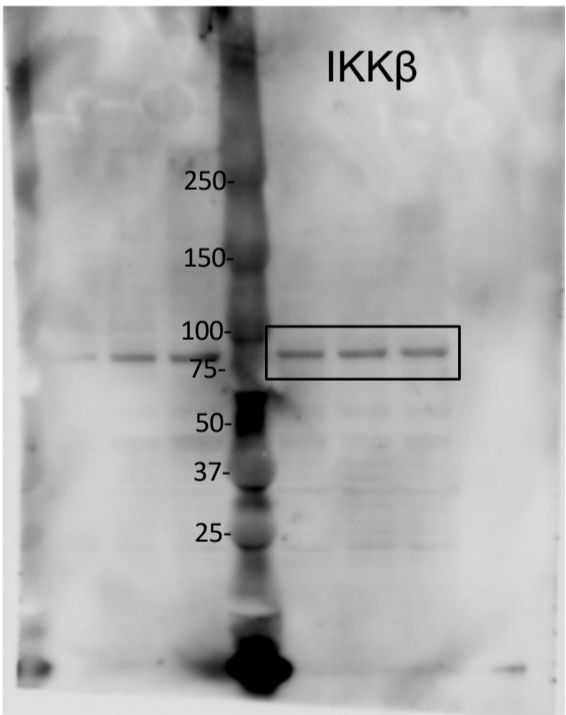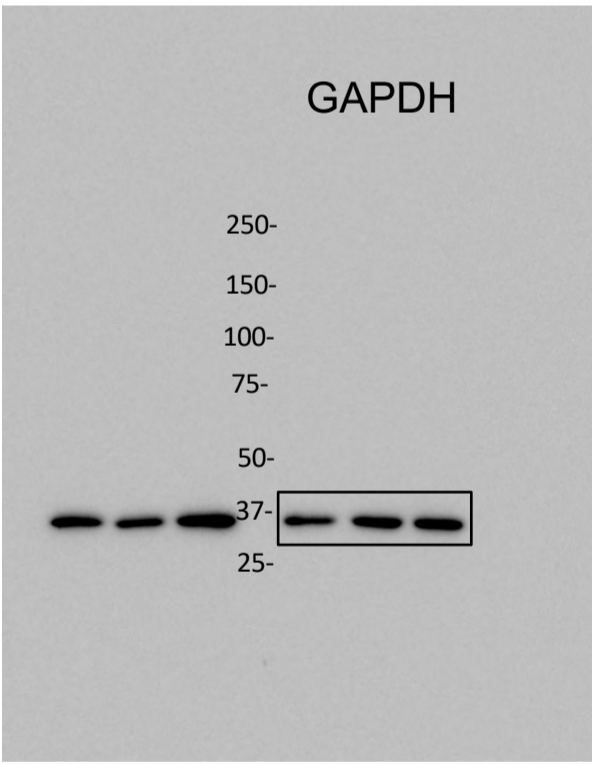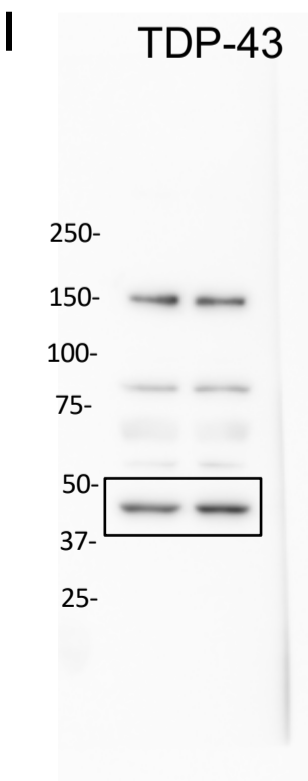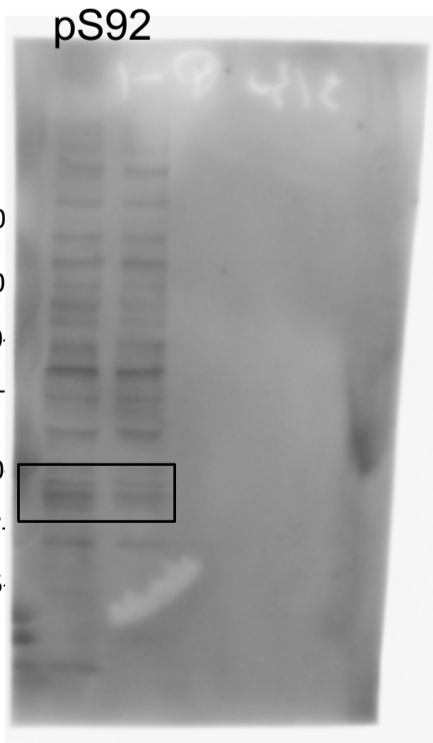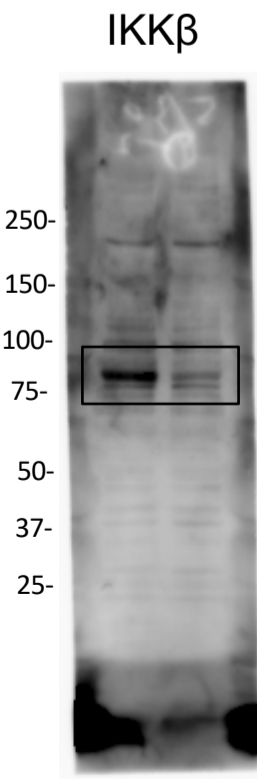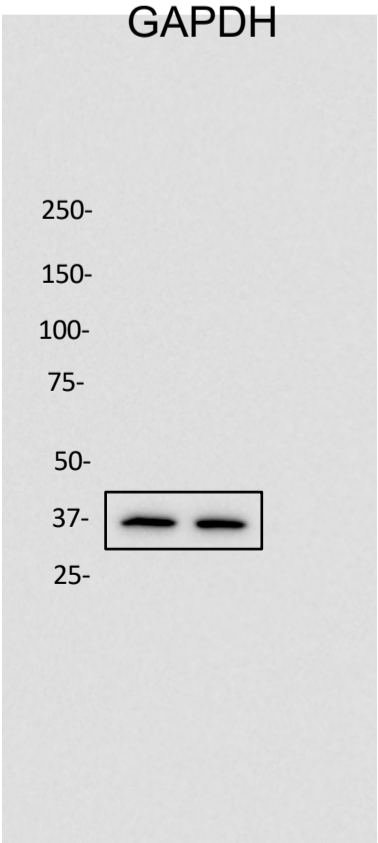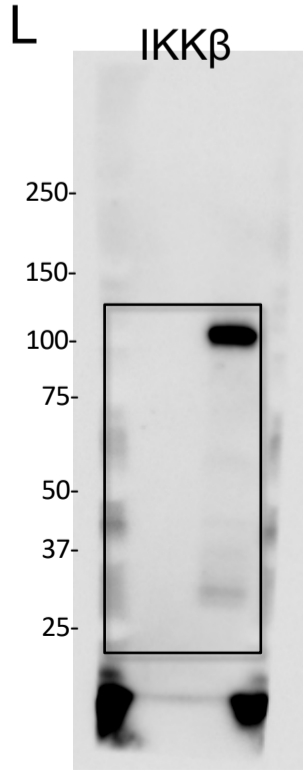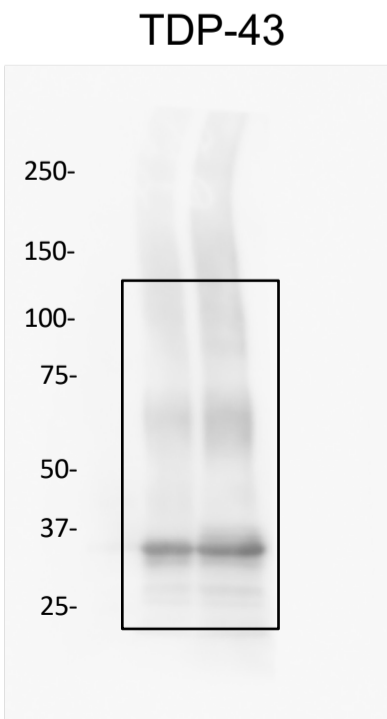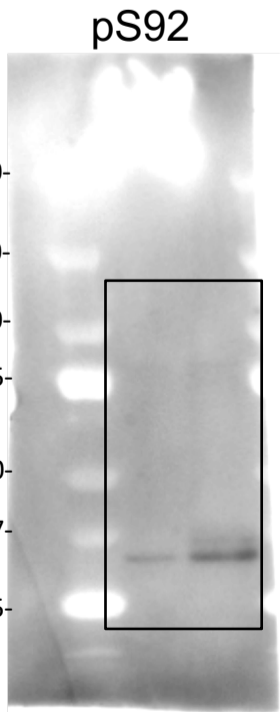

Supplement: SourceData F7 — is the source file for Fig. 7. [file JCB_202302048_SourceDataF7.pdf]

Figure S1

C

V5

GAPDH

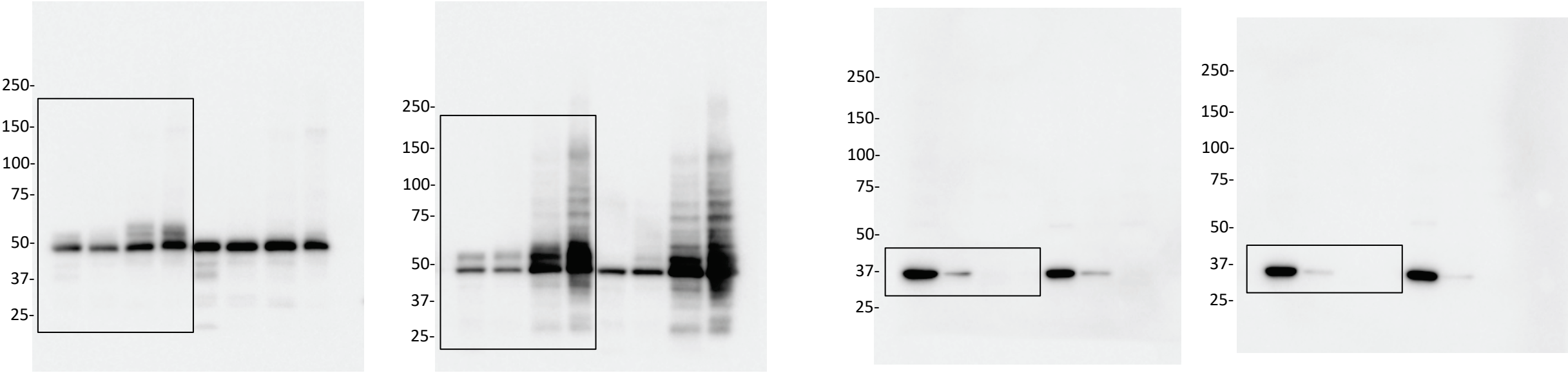

Supplement: SourceData FS1 — is the source file for Fig. S1. [file JCB_202302048_SourceDataFS1.pdf]

Figure S2

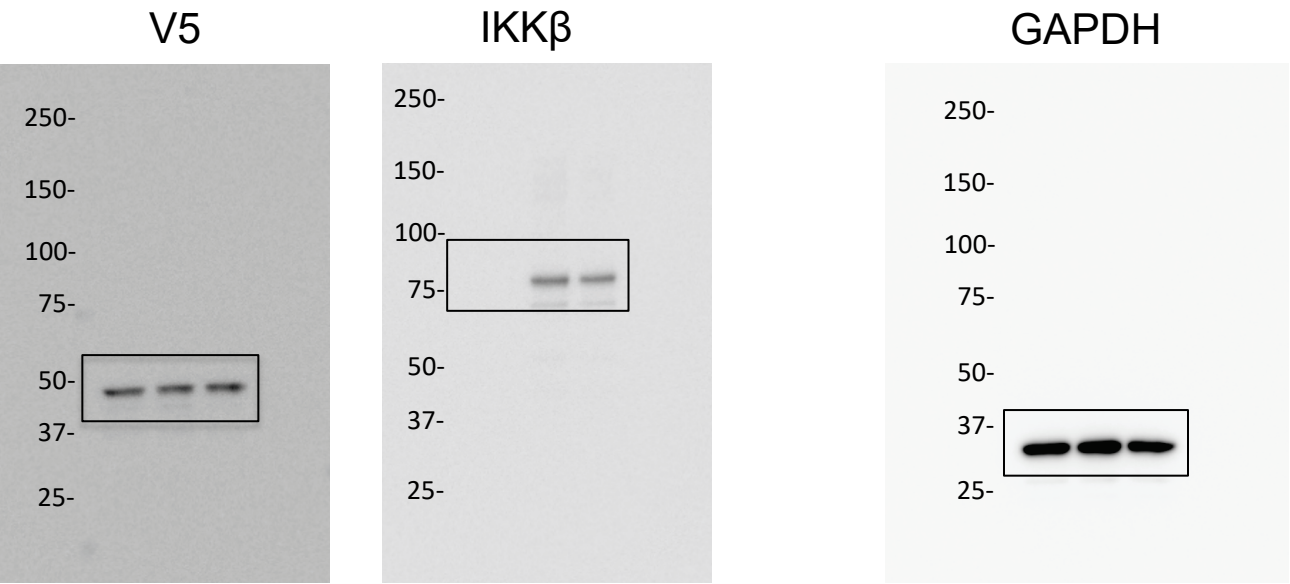

Supplement: SourceData FS2 — is the source file for Fig. S2. [file JCB_202302048_SourceDataFS2.pdf]

B

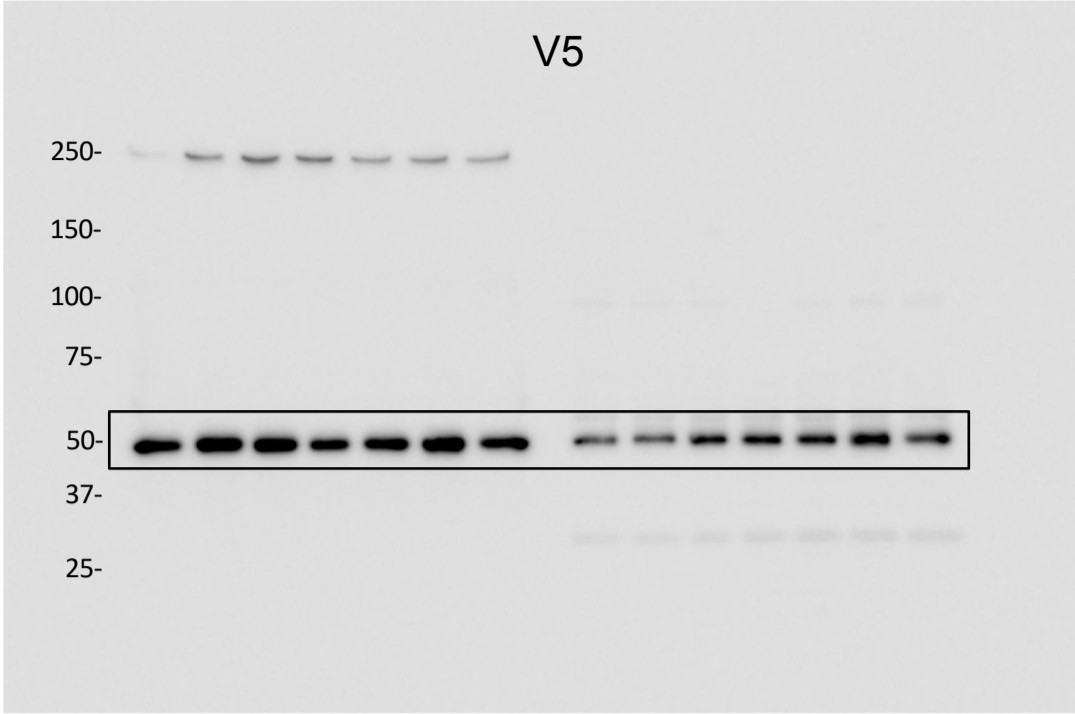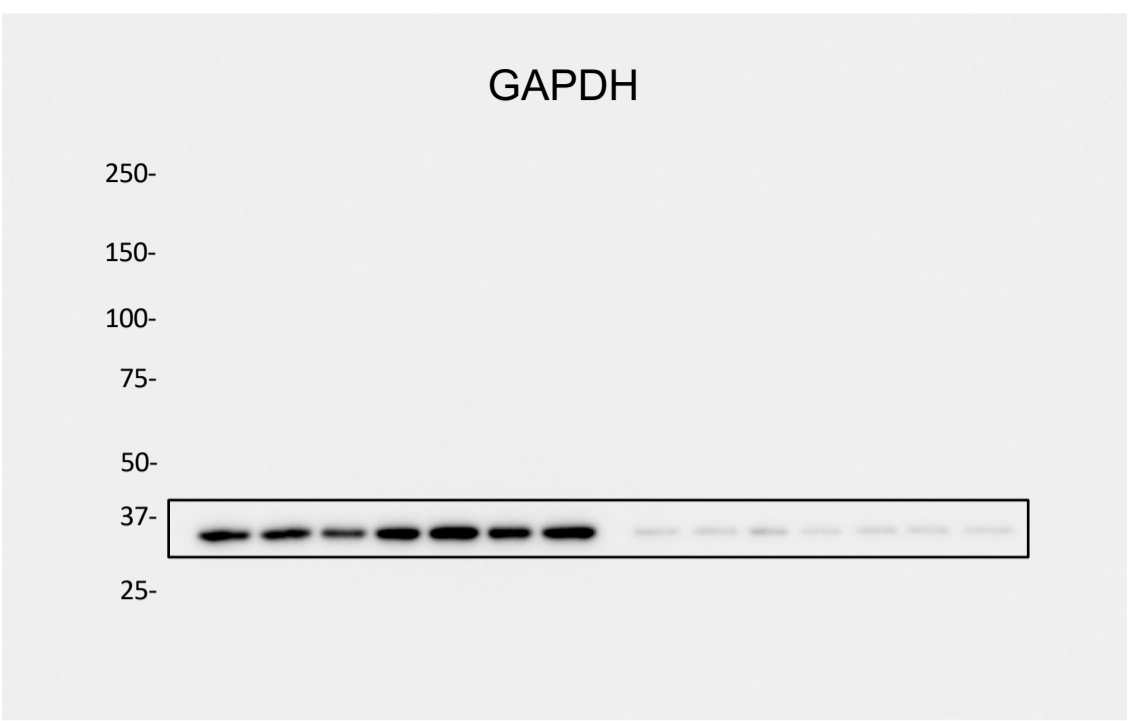

Supplement: SourceData FS3 — is the source file for Fig. S3. [file JCB_202302048_SourceDataFS3.pdf]
