# Supplementary material for: IκB kinase phosphorylates cytoplasmic TDP-43 and promotes its proteasome degradation
Source: J Cell Biol. 2024 Jan 10;223(2):e202302048. doi: 10.1083/jcb.202302048 (PMC10783433; doi:10.1083/jcb.202302048)
Supplement: SourceData F9 — is the source file for Fig. 9. [file JCB_202302048_SourceDataF9.pdf]

!

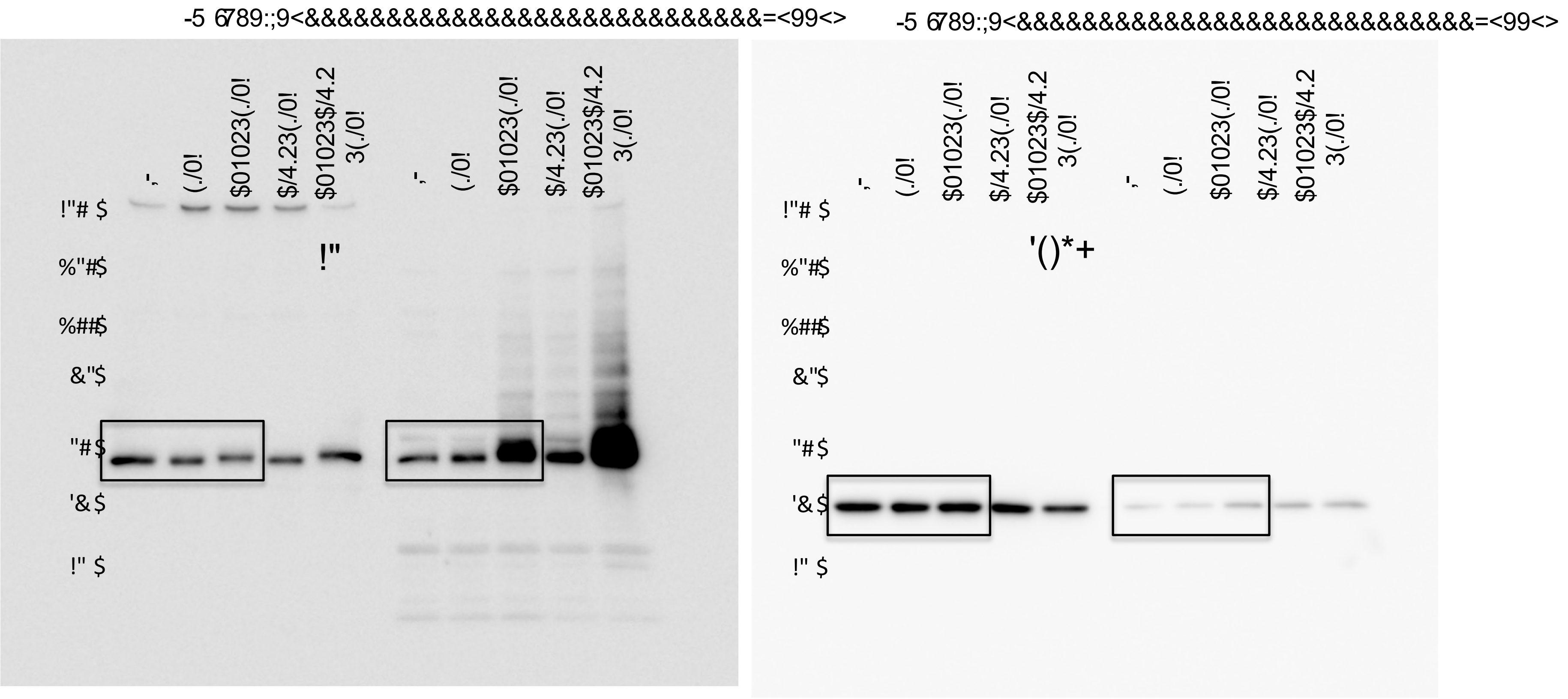
-5 6789:;9<&&&&&&&&&&&&&&&&&&&&&&&&&&&&&=<99<>
!"
'()*+

"

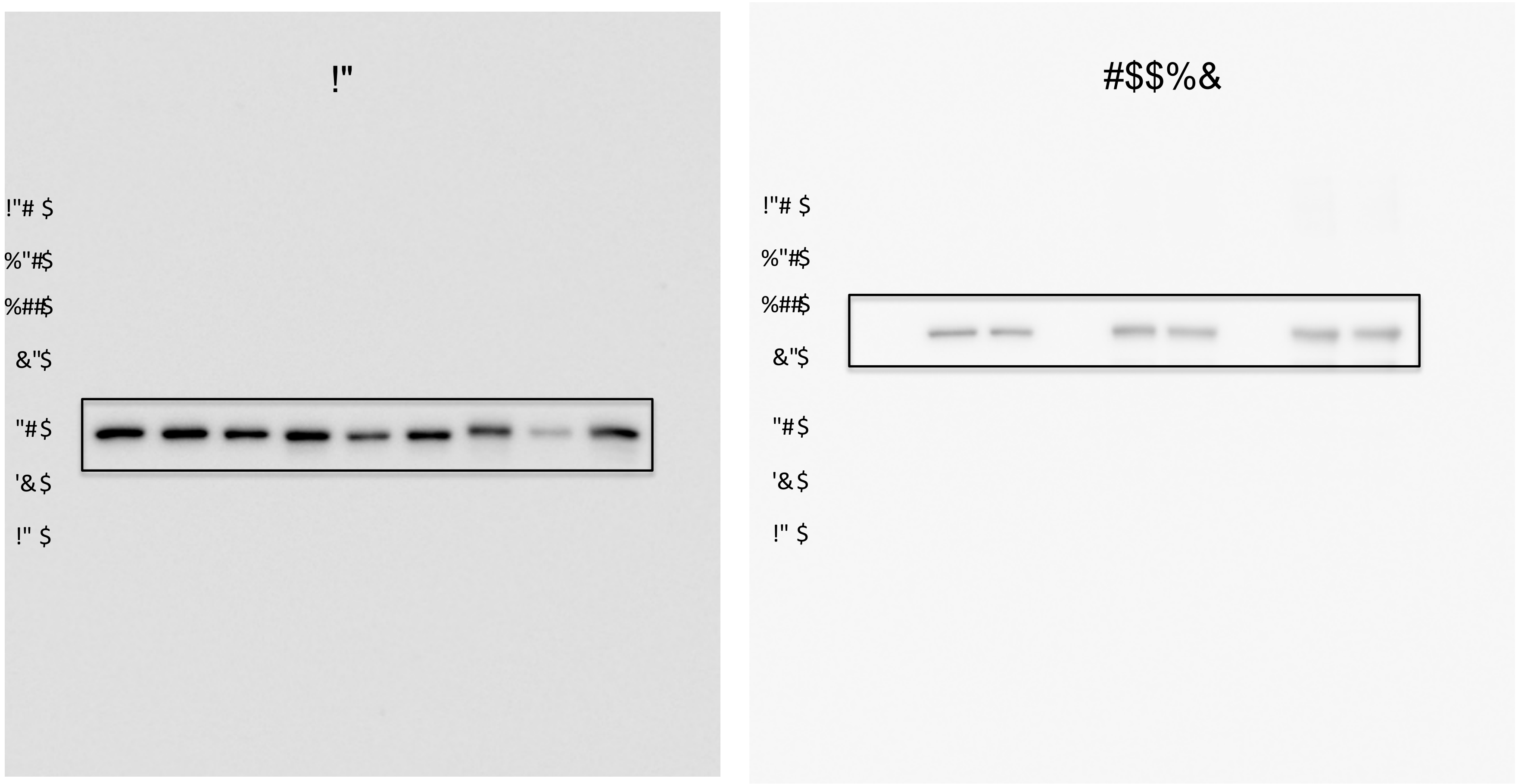
!"
#$$%&

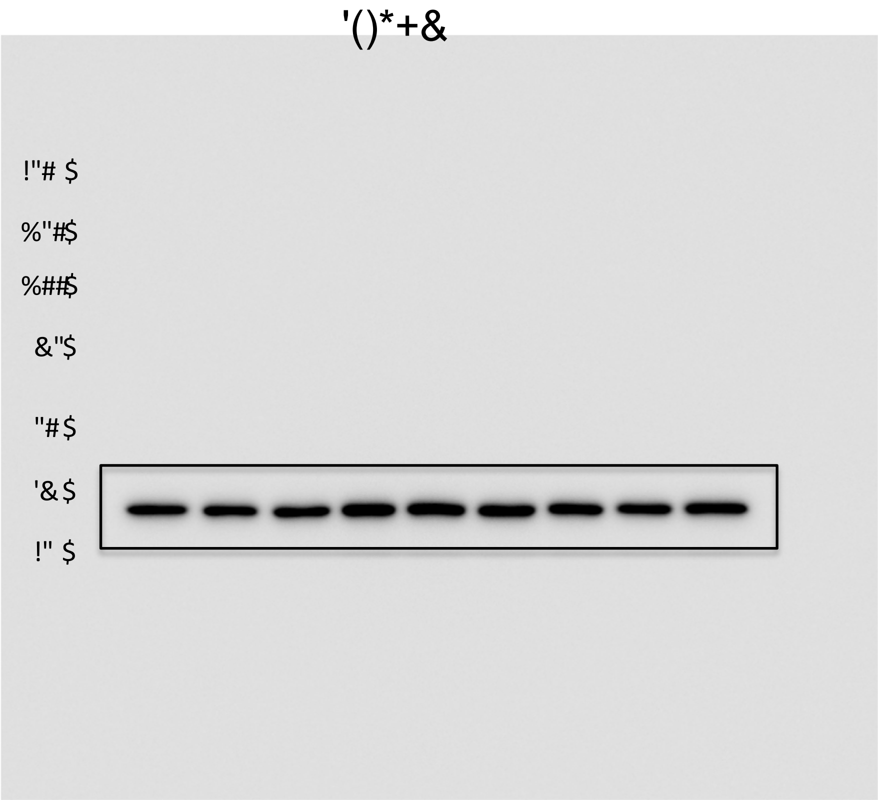
'()*+&
